# ARTS AND CREATIVITY AND THEIR IMPACT ON HEALTH AND WELL-BEING: A SYSTEMATIC REVIEW OF THE EVIDENCE

**DOI:** 10.1093/geroni/igac059.846

**Published:** 2022-12-20

**Authors:** Roger O'Sullivan, Laura McQuade

**Affiliations:** Institute of Public Health in Ireland, Belfast/Dublin, Not Applicable, Ireland; Institute of Public Health, Belfast, Northern Ireland, United Kingdom

## Abstract

**Aim:**

The aim of this mixed method systematic review was to develop a better understanding of the evidence on the impact of arts and creativity on the physical health and well-being of people aged 50+. Method: 73 studies were eligible for inclusion within the review and were appraised using the Mixed Methods Appraisal Tool.

**Results:**

Dance was associated with improved balance, lower body physical strength and flexibility, aerobic fitness. Promising evidence showed that music and singing were associated with improved cognitive function, quality of life, affective states, and a sense of well-being. Preliminary evidence showed that visual and creative arts were associated with reduced feelings of loneliness, improved sense of community and social connectedness. Initial evidence showed that theatre and drama were associated with emotional well-being.

**Conclusion:**

These findings highlight the value of participation in the arts and have implications for both public health and the arts and creativity agenda.

